# Giant Brunner’s gland adenoma as an unusual cause of anaemia: report of a case

**DOI:** 10.2478/v10019-010-0053-5

**Published:** 2010-12-31

**Authors:** Ali Coskun, Nazif Erkan

**Affiliations:** 1 Department of Surgery, Izmir Bozyaka Training and Research Hospital, Izmir, Turkey; 2 Department of Emergency Medicine, Izmir Bozyaka Training and Research Hospital, Izmir, Turkey

**Keywords:** Brunner’s gland, adenoma, anaemia

## Abstract

**Background:**

Brunner’s gland adenoma (BGA) is a rare benign duodenal tumour proliferating from Brunner’s glands. Here, we present a giant BGA leading to anaemia, with its clinical, endoscopic, radiological, surgical and pathological findings.

**Case report.:**

A 48-year-old Turkish man complained of a six months history of vague epigastric discomfort, loss of appetite and nausea after meals without vomiting. The physical examination had no unremarkable finding. Laboratory findings, including liver function tests, were within normal limits except a hypochromic, microcytic anaemia. The upper gastrointestinal endoscopic examination revealed a lobulated, red, polypoid tumour with a smooth surface covered with normal mucosa. The tumour was located on the anterior surface of duodenal bulb and had a wide base measuring 3.5 × 4 cm in size. Endoscopic ultrasonography revealed a submucosal polypoid mass located at the anterior surface of duodenal bulb. The endoscopic excision was tried but was not successful. The patient was operated and transduodenal polypectomy was done. The postoperative period was uneventful and the pathologic diagnosis was assessed as Brunner’s gland adenoma. During the follow-up period, the endoscopic examination was normal at 12th month postoperatively.

**Conclusions:**

BGA is a rare benign cause of anaemia that can be treated with excellent results.

## Introduction

Brunner’s gland adenoma (BGA), also known as Brunneroma or polypoid hamartoma, is a rare benign duodenal tumour proliferating from Brunner’s glands of duodenum. They are usually asymptomatic and discovered during endoscopy or on an upper gastrointestinal series.^1^

Here we present a giant BGA leading to anaemia, with its clinical, endoscopic, radiological, surgical and pathological findings.

## Case Report

**FIGURE 1.**
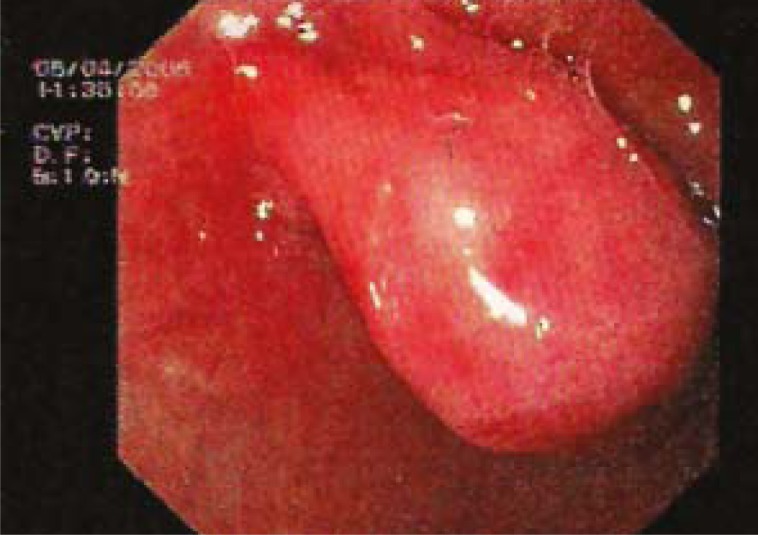
Upper GI endoscopic examination revealed a lobulated, red, polypoid tumour with a smooth surface covered with normal mucosa. The tumour located on the anterior surface of duodenal bulb, had a wide base, measuring 3.5 × 4 cm in size.

**FIGURE 2.**
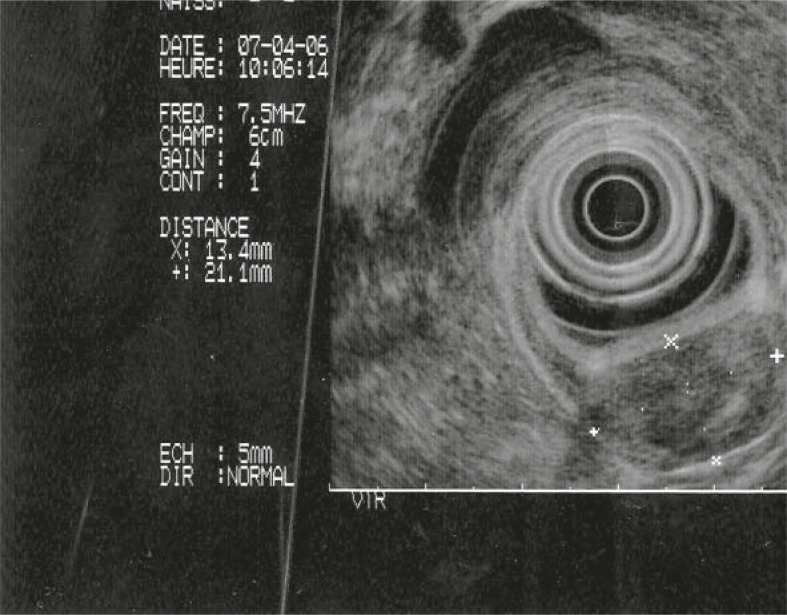
Endoscopic ultrasonography revealed a submucosal polypoid mass located at the anterior surface of duodenal bulb.

A 48-year-old Turkish man complained of a six months history of vague epigastric discomfort, loss of appetite and nausea after meals without vomiting. The physical examination had no unremarkable finding. Laboratory findings, including liver function tests, were within normal limits except a hypochromic, microcytic anaemia (Haemoglobin: 10 g/dl). The upper gastrointestinal endoscopic examination revealed a lobulated, red colour, polypoid tumour with a smooth surface covered with normal mucosa (Figure 1). The tumour located on the anterior surface of duodenal bulb had a wide base measuring 3.5 x 4 cm in size. Endoscopic ultrasonography revealed a submucosal polypoid mass located at the anterior surface of duodenal bulb (Figure 2).

**FIGURE 3.**
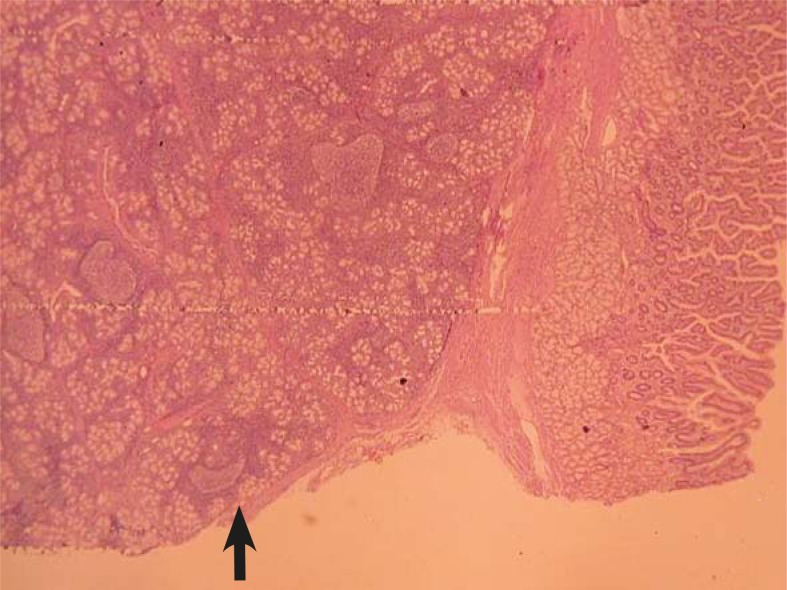
Light microscopy revealed hyperplasia of Brunner’s glands within the *lamina propria* of the duodenum (dark arrow) and normal duodenal mucosa (white arrow) (H&Ex40).

The endoscopic excision was tried but was not successful and biopsy was made and it was reported as gastric metaplasia. The patient was operated. A duodenotomy was performed and anteriorly located tumour was totally excised. The postoperative period was uneventful and the pathologic diagnosis was assessed as Brunner’s gland adenoma (Figure 3). During the follow-up period, the endoscopic examination was normal at the 12 month postoperatively. He has been followed without any symptom for four years.

## Discussion

Brunner’s glands are alkaline secreting glands in the submucosal layer of the duodenum. They are branched acinotubular structure. Brunner first described glands in duodenal tissue in 1688. They secrete mucus, urogastrone and pepsinogen and their primary function appears to protect the surface epithelium from acid chyme from stomach. The majority of glands are located in the first portion of the duodenum with decreasing prevalence in the second and third portion of duodenum.^1^,^2^

Benign tumours of the duodenum are very rare, with an incidence of 0.008% in a single autopsy study and those BGA comprise 10.6 % of these tumours.^3^,^4^ Since the original description of Brunner’s gland hamartoma in 1876 by Salvioli^5^, fewer than 200 cases have been reported in English literature.^6^ Various nomenclatures have been used to describe these tumours including Brunner’s gland hamartoma, adenoma and Brunneroma. As BGA grow, they typically form polypoid pedunculated masses. The pathogenesis of BGA remains unclear. It has been hypothesized that BGA is related to hyperacidity with compensatory growth of the alkaline-secreting Brunner’s gland, or to Helicobacter pylori infection.^2^,^6^ BGA has equivalent gender and race distribution with age of presentation typically in the fifth or sixth decade of life. BGA is often an incidental finding during esophago-gastro-duodenoscopy or imaging studies as majority of patients are asymptomatic. In these patients tumour tends to be smaller, which may account for their absence of symptoms.

In symptomatic patients, BGA presents with hemorrhagic or obstructive symptoms. A group of patients presented with tumour-related blood loss, which, in the majority, is chronic and does not result in hemodynamic instability. The BGA could lead to a gross upper gastrointestinal bleeding due to ulceration of the mucosa especially in tumours located in the first portion of the duodenum.^7^ Another group of patients with BGA presented with prolonged histories of obstructive upper gastrointestinal symptoms such as epigastric pain, bloating and early satiety. Rare presentations include obstructive jaundice and intussusception due to localization and size of the tumour.^8^ In our patient, BGA is located in the first portion of duodenum, and leads to dyspeptic symptoms with anaemia due to a chronic blood loss.

The diagnosis of this lesion can be made like in other intestinal tumours radiologically or endoscopically.^9^ Before endoscopy, small bowel series were the main tool for the diagnosis. The pedunculated BGA is featured by a well-defined smooth and round filling defect, whereas the nodular and diffuse varieties are seen as multiple filling defects in the duodenum, described as “Swiss cheese” in appearance. The description of BGA on computed tomography has been limited but varies from homogenous enhancement with intravenous contrast administration to heterogeneous lesions with solid and cystic components.^10^ The endoscopic US examination clearly demonstrates heterogeneous lesions with solid and cystic components.^11^ Endoscopy allows a direct visualization of the lesion, biopsy to rule out malignancy and the option of the endoscopic resection. Biopsies are typically indeterminate given the submucosal location of the lesion.^5^,^6^,^9^ In our case, all diagnostic tools including upper gastrointestinal endoscopy and endoscopic US indicated a submucosal polypoid mass.

The pathologic features of these tumours are characteristic. The lack of dysplasia, unusual admixture of normal tissues including Brunner’s glands, ducts, adipose tissue and lymphoid tissue consist the texture of pathology.^2^ The differential diagnosis includes duplication cyst, leiomyoma, leiomyosarcoma, adenoma or adenocarcinoma, lymphoma, carcinoid tumours, heterotopic pancreatic or gastric tissue or gastrointestinal stromal tumours.^1^,^2^,^6^

The treatment can be different according to size, symptoms and suspicious of malignancy. The conservative management is advocated for asymptomatic diffuse hyperplasia because it is considered to have no neoplastic potential. Symptomatic and larger lesions leading to bleeding or obstruction should be excised either endoscopically or surgically. The endoscopic treatment is especially useful for pedunculated lesions. However, if endoscopic interventions fail, the surgical resection may be necessary in symptomatic patients or those in whom a malignancy is suspected.^2^,^7^ In our case, the endoscopic removal was unsuccessful since the tumour was huge in size and in a broad wide-base. Then we made a transduodenal polpectomy.

In conclusion, BGA is a rare benign cause of anaemia that could be treated by the endoscopic or the surgical resection with an excellent outcome.
